# (*E*)-4-[4-(Dimethyl­amino)benzyl­ideneamino]benzonitrile

**DOI:** 10.1107/S1600536809047023

**Published:** 2009-11-14

**Authors:** Jian-Cheng Zhou, Zheng-Yun Zhang, Nai-Xu Li, Chuan-Ming Zhang

**Affiliations:** a College of Chemistry and Chemical Engineering, Southeast University, Nanjing 211189, People’s Republic of China; b Jiangsu Provincial Key Laboratory of Pulp and Paper Science and Technology, Nanjing Forestry University, Nanjing 210037, People’s Republic of China

## Abstract

The mol­ecule of the title compound, C_16_H_15_N_3_, displays a *trans* configuration with respect to the C=N double bond. The mol­ecule is not planar, the dihedral angle between the benzene rings being 57.83 (9)°. The crystal packing is stabilized only by van der Waals inter­actions.

## Related literature

For the pharmacological activity of Schiff base compounds, see: Zhou *et al.* (2000 ▶); Sriram *et al.* (2006 ▶). For reference structural data, see: Allen *et al.* (1987 ▶).
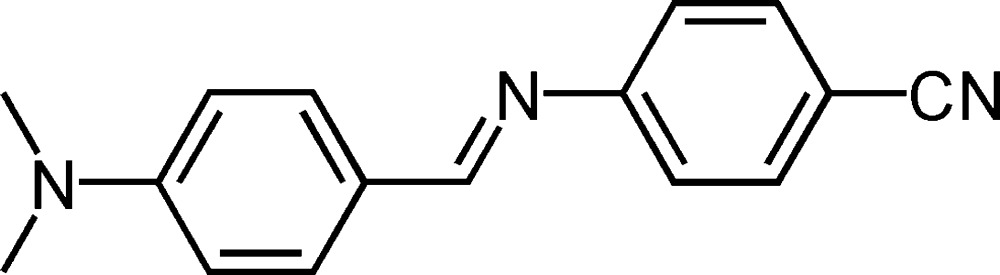


## Experimental

### 

#### Crystal data

C_16_H_15_N_3_
*M* *_r_* = 249.31
Monoclinic, 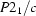

*a* = 9.733 (6) Å
*b* = 16.159 (9) Å
*c* = 9.103 (6) Å
β = 110.644 (12)°
*V* = 1339.8 (14) Å^3^
*Z* = 4
Mo *K*α radiation
μ = 0.08 mm^−1^
*T* = 293 K
0.20 × 0.20 × 0.20 mm

#### Data collection

Rigaku SCXmini diffractometer
Absorption correction: multi-scan (*CrystalClear*; Rigaku, 2005 ▶) *T*_min_ = 0.985, *T*_max_ = 0.985
13048 measured reflections
2610 independent reflections
2134 reflections with *I* > 2σ(*I*)
*R*_int_ = 0.043

#### Refinement

*R*[*F* ^2^ > 2σ(*F* ^2^)] = 0.070
*wR*(*F* ^2^) = 0.221
*S* = 1.09
2610 reflections
172 parameters
H-atom parameters constrained
Δρ_max_ = 0.53 e Å^−3^
Δρ_min_ = −0.36 e Å^−3^

### 

Data collection: *CrystalClear* (Rigaku, 2005 ▶); cell refinement: *CrystalClear*; data reduction: *CrystalClear*; program(s) used to solve structure: *SHELXS97* (Sheldrick, 2008 ▶); program(s) used to refine structure: *SHELXL97* (Sheldrick, 2008 ▶); molecular graphics: *SHELXTL* (Sheldrick, 2008 ▶); software used to prepare material for publication: *SHELXL97*.

## Supplementary Material

Crystal structure: contains datablocks I, global. DOI: 10.1107/S1600536809047023/rz2389sup1.cif

Structure factors: contains datablocks I. DOI: 10.1107/S1600536809047023/rz2389Isup2.hkl

Additional supplementary materials:  crystallographic information; 3D view; checkCIF report

## Figures and Tables

**Fig. 1.**
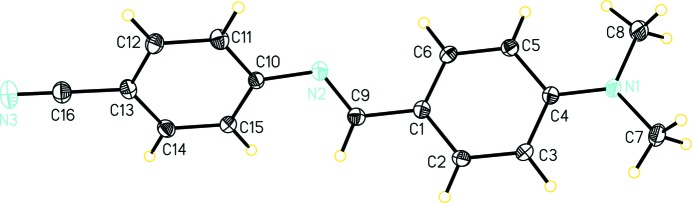
The molecular structure of the title compound, showing the atomic numbering scheme. The displacement ellipsoids are drawn at the 30% probability level.

## supplementary crystallographic information

### Comment

Schiff bases compounds are of great interest in many fields of chemistry and biochemistry, primarily due to significant pharmacological activities, *e. g.* anticancer (Zhou *et al.*, 2000) and anti-HIV (Sriram *et al.*, 2006) activities. In addition, Schiff base compounds play an important role in the development of coordination chemistry related to magnetism and catalysis. Here, we report the synthesis and crystal structure of the title compound.

The molecular structure of the the title compound is shown in Fig. 1. All bond lengths (Allen *et al.*, 1987) and angles in the molecule are normal. The N=C bond distance is 1.288 (3) Å. The structure displays a *trans* configuration about the central C9=N2 double bond. The molecule is not planar, as indicated by the dihedral angle between the two benzene rings of 57.83 (9)°. The crystal packing is stabilized only by van der Waals interactions.

### Experimental

A solution of 4-(dimethylamino)benzaldehyde (0.596 g, 4 mmol) in ethanol (20 ml) was added to a solution of 4-aminobenzonitrile (0.472 g, 4 mmol) in methanol (20 ml), and the mixture stirred for 6 h under reflux. The resulting yellow precipitate was filtered off and crystals of the title compound suitable for X-ray analysis were obtained by slow evaporation of an ethanol solution.

### Refinement

All the H atoms were located geometrically and treated as riding atoms with C—H = 0.93–0.96 Å, and with *U*_iso_(H) = 1.2 *U*_eq_(C) or 1.5 *U*_eq_(C) for methyl H atoms.

### Crystal data

**Table:**

C_16_H_15_N_3_	*F*(000) = 528
*M**_r_* = 249.31	*D*_x_ = 1.236 Mg m^−^^3^
Monoclinic, *P*2_1_/*c*	Mo *K*α radiation, λ = 0.71073 Å
Hall symbol: -P 2ybc	Cell parameters from 3347 reflections
*a* = 9.733 (6) Å	θ = 2.2–27.5°
*b* = 16.159 (9) Å	µ = 0.08 mm^−^^1^
*c* = 9.103 (6) Å	*T* = 293 K
β = 110.644 (12)°	Block, yellow
*V* = 1339.8 (14) Å^3^	0.20 × 0.20 × 0.20 mm
*Z* = 4	

### Data collection

**Table:**

Rigaku SCXmini diffractometer	2610 independent reflections
Radiation source: fine-focus sealed tube	2134 reflections with *I* > 2σ(*I*)
graphite	*R*_int_ = 0.043
Detector resolution: 13.6612 pixels mm^-1^	θ_max_ = 26.0°, θ_min_ = 2.2°
ω scans	*h* = −12→12
Absorption correction: multi-scan (*CrystalClear*; Rigaku, 2005)	*k* = −19→19
*T*_min_ = 0.985, *T*_max_ = 0.985	*l* = −11→11
13048 measured reflections	

### Refinement

**Table:**

Refinement on *F*^2^	Primary atom site location: structure-invariant direct methods
Least-squares matrix: full	Secondary atom site location: difference Fourier map
*R*[*F*^2^ > 2σ(*F*^2^)] = 0.070	Hydrogen site location: inferred from neighbouring sites
*wR*(*F*^2^) = 0.221	H-atom parameters constrained
*S* = 1.09	*w* = 1/[σ^2^(*F*_o_^2^) + (0.1227*P*)^2^ + 0.7975*P*] where *P* = (*F*_o_^2^ + 2*F*_c_^2^)/3
2610 reflections	(Δ/σ)_max_ < 0.001
172 parameters	Δρ_max_ = 0.53 e Å^−^^3^
0 restraints	Δρ_min_ = −0.36 e Å^−^^3^

### Special details

**Table:**

Geometry. All e.s.d.'s (except the e.s.d. in the dihedral angle between two l.s. planes) are estimated using the full covariance matrix. The cell e.s.d.'s are taken into account individually in the estimation of e.s.d.'s in distances, angles and torsion angles; correlations between e.s.d.'s in cell parameters are only used when they are defined by crystal symmetry. An approximate (isotropic) treatment of cell e.s.d.'s is used for estimating e.s.d.'s involving l.s. planes.
Refinement. Refinement of *F*^2^ against ALL reflections. The weighted *R*-factor *wR* and goodness of fit *S* are based on *F*^2^, conventional *R*-factors *R* are based on *F*, with *F* set to zero for negative *F*^2^. The threshold expression of *F*^2^ > σ(*F*^2^) is used only for calculating *R*-factors(gt) *etc*. and is not relevant to the choice of reflections for refinement. *R*-factors based on *F*^2^ are statistically about twice as large as those based on *F*, and *R*- factors based on ALL data will be even larger.

### Fractional atomic coordinates and isotropic or equivalent isotropic displacement parameters (Å^2^)

**Table:**

	*x*	*y*	*z*	*U*_iso_*/*U*_eq_	
N2	0.9054 (2)	0.11253 (12)	0.0481 (2)	0.0259 (5)	
N1	1.3132 (2)	0.12345 (13)	0.7796 (2)	0.0266 (5)	
C4	1.2390 (3)	0.13388 (14)	0.6221 (3)	0.0224 (5)	
C14	0.7447 (3)	0.19677 (16)	−0.3581 (3)	0.0279 (6)	
H14A	0.7262	0.2472	−0.4105	0.033*	
C15	0.8167 (3)	0.19445 (15)	−0.1968 (3)	0.0254 (5)	
H15A	0.8479	0.2435	−0.1415	0.030*	
C9	1.0202 (3)	0.15527 (14)	0.1238 (3)	0.0246 (5)	
H9A	1.0606	0.1897	0.0678	0.030*	
C10	0.8427 (2)	0.11873 (14)	−0.1164 (3)	0.0222 (5)	
C12	0.7285 (3)	0.04706 (15)	−0.3620 (3)	0.0278 (6)	
H12A	0.7008	−0.0022	−0.4174	0.033*	
C11	0.7979 (3)	0.04566 (15)	−0.2013 (3)	0.0280 (6)	
H11A	0.8151	−0.0048	−0.1487	0.034*	
C6	1.0225 (3)	0.11201 (14)	0.3895 (3)	0.0247 (5)	
H6A	0.9282	0.0907	0.3439	0.030*	
C16	0.6232 (3)	0.12454 (17)	−0.6090 (3)	0.0344 (6)	
C13	0.7000 (3)	0.12305 (15)	−0.4417 (3)	0.0264 (6)	
C5	1.0934 (3)	0.10420 (15)	0.5487 (3)	0.0249 (5)	
H5A	1.0456	0.0792	0.6092	0.030*	
C2	1.2299 (3)	0.18366 (15)	0.3669 (3)	0.0260 (6)	
H2B	1.2749	0.2115	0.3066	0.031*	
C3	1.3042 (3)	0.17532 (15)	0.5260 (3)	0.0265 (6)	
H3A	1.3983	0.1971	0.5708	0.032*	
C1	1.0889 (3)	0.15156 (14)	0.2934 (3)	0.0235 (5)	
N3	0.5616 (3)	0.12575 (18)	−0.7415 (3)	0.0523 (8)	
C8	1.2478 (3)	0.0794 (2)	0.8776 (3)	0.0427 (7)	
H8A	1.1447	0.0721	0.8211	0.064*	
H8B	1.2615	0.1108	0.9713	0.064*	
H8C	1.2939	0.0263	0.9053	0.064*	
C7	1.4701 (3)	0.14047 (19)	0.8465 (3)	0.0350 (6)	
H7A	1.5001	0.1699	0.7713	0.052*	
H7B	1.5231	0.0892	0.8729	0.052*	
H7C	1.4904	0.1735	0.9395	0.052*	

### Atomic displacement parameters (Å^2^)

**Table:**

	*U*^11^	*U*^22^	*U*^33^	*U*^12^	*U*^13^	*U*^23^
N2	0.0267 (10)	0.0271 (10)	0.0214 (10)	0.0019 (8)	0.0054 (9)	0.0007 (8)
N1	0.0246 (11)	0.0340 (12)	0.0196 (10)	−0.0042 (8)	0.0058 (8)	−0.0022 (8)
C4	0.0236 (12)	0.0212 (11)	0.0222 (11)	0.0013 (9)	0.0078 (9)	−0.0028 (9)
C14	0.0284 (13)	0.0287 (13)	0.0262 (13)	0.0007 (10)	0.0090 (10)	0.0048 (10)
C15	0.0266 (12)	0.0251 (12)	0.0238 (12)	−0.0028 (9)	0.0080 (10)	−0.0008 (9)
C9	0.0244 (12)	0.0227 (12)	0.0263 (12)	0.0026 (9)	0.0084 (10)	0.0023 (9)
C10	0.0184 (11)	0.0278 (12)	0.0203 (11)	0.0004 (9)	0.0067 (9)	0.0014 (9)
C12	0.0284 (13)	0.0276 (13)	0.0268 (13)	0.0012 (10)	0.0091 (10)	−0.0042 (10)
C11	0.0308 (13)	0.0245 (12)	0.0275 (13)	0.0066 (10)	0.0087 (10)	0.0037 (9)
C6	0.0199 (11)	0.0263 (12)	0.0269 (12)	−0.0012 (9)	0.0071 (10)	−0.0025 (9)
C16	0.0346 (14)	0.0389 (15)	0.0283 (15)	0.0001 (11)	0.0093 (12)	−0.0003 (11)
C13	0.0234 (12)	0.0340 (14)	0.0220 (12)	0.0031 (10)	0.0083 (10)	0.0018 (9)
C5	0.0251 (12)	0.0266 (12)	0.0246 (12)	−0.0018 (9)	0.0109 (10)	−0.0020 (9)
C2	0.0261 (12)	0.0252 (12)	0.0276 (12)	−0.0024 (10)	0.0103 (10)	0.0031 (9)
C3	0.0232 (12)	0.0279 (12)	0.0258 (12)	−0.0052 (9)	0.0055 (10)	−0.0016 (9)
C1	0.0244 (12)	0.0211 (11)	0.0237 (12)	0.0017 (9)	0.0066 (10)	−0.0011 (9)
N3	0.0589 (18)	0.0662 (19)	0.0246 (13)	−0.0025 (14)	0.0058 (12)	−0.0003 (11)
C8	0.0412 (16)	0.063 (2)	0.0232 (13)	−0.0160 (14)	0.0105 (12)	0.0014 (12)
C7	0.0250 (13)	0.0527 (17)	0.0237 (12)	−0.0017 (11)	0.0042 (10)	−0.0014 (11)

### Geometric parameters (Å, °)

**Table:**

N2—C9	1.288 (3)	C11—H11A	0.9300
N2—C10	1.408 (3)	C6—C5	1.374 (3)
N1—C4	1.370 (3)	C6—C1	1.411 (3)
N1—C8	1.452 (3)	C6—H6A	0.9300
N1—C7	1.457 (3)	C16—N3	1.143 (4)
C4—C3	1.417 (3)	C16—C13	1.441 (3)
C4—C5	1.420 (3)	C5—H5A	0.9300
C14—C15	1.387 (3)	C2—C3	1.379 (3)
C14—C13	1.398 (4)	C2—C1	1.397 (3)
C14—H14A	0.9300	C2—H2B	0.9300
C15—C10	1.402 (3)	C3—H3A	0.9300
C15—H15A	0.9300	C8—H8A	0.9600
C9—C1	1.451 (3)	C8—H8B	0.9600
C9—H9A	0.9300	C8—H8C	0.9600
C10—C11	1.394 (3)	C7—H7A	0.9600
C12—C11	1.378 (3)	C7—H7B	0.9600
C12—C13	1.403 (3)	C7—H7C	0.9600
C12—H12A	0.9300		
			
C9—N2—C10	120.0 (2)	N3—C16—C13	179.6 (3)
C4—N1—C8	121.3 (2)	C14—C13—C12	119.8 (2)
C4—N1—C7	120.2 (2)	C14—C13—C16	120.5 (2)
C8—N1—C7	117.2 (2)	C12—C13—C16	119.7 (2)
N1—C4—C3	121.3 (2)	C6—C5—C4	120.8 (2)
N1—C4—C5	121.4 (2)	C6—C5—H5A	119.6
C3—C4—C5	117.3 (2)	C4—C5—H5A	119.6
C15—C14—C13	119.8 (2)	C3—C2—C1	122.0 (2)
C15—C14—H14A	120.1	C3—C2—H2B	119.0
C13—C14—H14A	120.1	C1—C2—H2B	119.0
C14—C15—C10	120.4 (2)	C2—C3—C4	120.7 (2)
C14—C15—H15A	119.8	C2—C3—H3A	119.6
C10—C15—H15A	119.8	C4—C3—H3A	119.6
N2—C9—C1	122.3 (2)	C2—C1—C6	117.3 (2)
N2—C9—H9A	118.9	C2—C1—C9	120.0 (2)
C1—C9—H9A	118.9	C6—C1—C9	122.6 (2)
C11—C10—C15	119.1 (2)	N1—C8—H8A	109.5
C11—C10—N2	117.6 (2)	N1—C8—H8B	109.5
C15—C10—N2	123.2 (2)	H8A—C8—H8B	109.5
C11—C12—C13	119.8 (2)	N1—C8—H8C	109.5
C11—C12—H12A	120.1	H8A—C8—H8C	109.5
C13—C12—H12A	120.1	H8B—C8—H8C	109.5
C12—C11—C10	121.0 (2)	N1—C7—H7A	109.5
C12—C11—H11A	119.5	N1—C7—H7B	109.5
C10—C11—H11A	119.5	H7A—C7—H7B	109.5
C5—C6—C1	121.7 (2)	N1—C7—H7C	109.5
C5—C6—H6A	119.1	H7A—C7—H7C	109.5
C1—C6—H6A	119.1	H7B—C7—H7C	109.5
